# Comparison of retention and denture base adaptation between conventional and 3D-printed complete dentures

**DOI:** 10.34172/joddd.2022.030

**Published:** 2022-11-15

**Authors:** Radwa Mohsen Kamal Emera, Mohamed Shady, Mahmoud Almabrouk Alnajih

**Affiliations:** ^1^Prosthodontics Department, Faculty of Dentistry, Mansoura University, Mansoura, Egypt; ^2^Faculty of Dentistry, Zawia University, Libya

**Keywords:** 3D printing, CAD-CAM, Complete dentures, Denture retention

## Abstract

**Background.** In recent years, complete denture construction has been revolutionized using computer-aided designing and computer-aided manufacturing (CAD-CAM) technology. This clinical study compared the retention and denture base adaptation of 3D-printed complete dentures fabricated using dimethacrylate-based resins with a photoinitiator versus conventional complete dentures.

**Methods.** Ten completely edentulous patients were nominated for this study. Each patient received two complete dentures. One complete denture was conventionally fabricated, while the other was digitally fabricated through 3D printing of dimethacrylate-based resins with a photoinitiator. Denture base adaptation for both dentures was evaluated using surface matching software. Denture retention was also evaluated using a digital force meter at the time of complete denture insertion (T_0_ ), after three months (T_3_ ) and six months (T_6_ ) of denture use.

**Results.** Regarding adaptation (mean misfit values), a statistically insignificant difference was detected between the two differently fabricated complete dentures. In addition, comparable satisfactory retention values were observed for both conventionally fabricated and 3D-printed dentures; however, there was an insignificant increase in retention over time.

**Conclusion.** 3D-printed complete dentures fabricated using dimethacrylate-based resins could be a promising alternative to conventionally fabricated complete dentures with satisfactory denture base adaptation and retention.

## Introduction

 Accurately adapted denture bases are necessary for achieving acceptable retention and stability for complete dentures. Numerous methods are available for manufacturing complete dentures. The worthy aim of each method is to achieve an equilibrium between biocompatibility, adaptation, minimum deformation, and esthetics.^1,2^

 Since 1936, many processing procedures have been established and successfully used in traditional procedures to construct complete dentures. It does, though, necessitate a time-consuming series of many laboratory and clinical steps. Poly-methyl methacrylate (PMMA) polymerization shrinkage and the inconvenience of creating a duplicate denture are assumed disadvantages of that approach.^3^

 A new CAD-CAM (computer-aided designing and computer-aided manufacturing) technology has been introduced to fabricate complete dentures. CAD/CAM refers to using computers to assist in product designing and manufacturing. Additive and subtractive manufacturing processes are included. Through additive manufacturing (3-dimensional printing), digital images are employed to generate a product by laying down successive layers of powder or liquid materials that are solidified afterward by exposure to UV light (photopolymerization). However, in subtractive manufacturing, digital images are used to fabricate a product by milling a prefabricated block to achieve the required geometry.^4-6^

 CAD-CAM production of complete dentures is distinguished by simplicity and decreased laboratory steps. It also offers superior dimensional precision and standardized production.^7,8^ Digital design confirms a regular thickness of denture base that can be modified and maintained minimal to ensure patient comfort. Furthermore, the digital data allow future construction of dentures if necessary.^9^

 Goodacre et al^10^ evaluated the precision and duplicability of milled dentures compared to the traditionally fabricated ones. The milled dentures outperformed the traditionally constructed ones. Hwang et al^11^ compared the trueness and adaptability of conventional, milled, and printed denture bases and found that the printed bases performed better.

 Further clinical studies are needed to assess the various features of CAD-CAM dentures. Consequently, this study aimed to compare 3D-printed denture bases to traditional heat-polymerized dentures regarding retention and denture base adaptation. The initial null hypothesis was that there would be no difference in the intaglio surface adaptation of the denture bases to the edentulous maxillary and mandibular casts across the two manufacturing procedures. The second null hypothesis was that there would be no difference in denture retention between the two procedures.

## Methods

 Ten completely edentulous patients aged 45‒60 were selected for this study. All the study procedures were clarified to the patients. The research protocol was accepted by the Faculty of Dentistry, Mansoura University’s Research Ethics Committee. All the patients were healthy, free from systemic diseases, and exhibited good oral hygiene. They had completely edentulous jaws for at least one year, with no previous denture experience. Residual alveolar ridges were covered with healthy, firm mucosa, free of tissue undercuts. All the patients had Angle’s class I maxillo-mandibular relationship.

###  Study design 

 A crossover design was conducted. Each patient received two types of prostheses. This design allowed the standardization of factors affecting the results. Furthermore, each patient served as a control for himself. The same operator constructed all dentures.

 Each patient received two prostheses; conventional and 3D-printed complete dentures. The succession of complete denture insertion was randomized to diminish the effect of the order of complete denture use on the results. First, each denture was used for three months, followed by two weeks of rest; then, the other denture type was delivered to the patient and used for another three months.

###  Construction of conventional complete dentures

 A clinical protocol was used to fabricate CDs. The various clinical procedures in the respective visits are described below:

 Irreversible hydrocolloid impression material (Alginate Cavex, Holland, normal set impression material) was used for making primary impressions, while the final impressions were made using a zinc oxide eugenol-free impression material (Cavex Outline, Cavex Co., Holland). The final impressions were poured to create the master casts. On the master cast, wax record blocks were constructed. The orientation relation was recorded for mounting the maxillary cast on a semi-adjustable articulator (Whipmix Semi-adjustable Articulator) using a facebow record. Then the mandibular cast was mounted using the intermaxillary jaw relation records. After mounting, acrylic artificial teeth were arranged for bilateral balanced occlusion, followed by intraoral try-in. Complete dentures were manufactured through conventional flasking procedures (Acroston, heat-cured acrylic resin, Egypt), finished, and polished. The finished dentures were remounted in the laboratory to correct occlusal discrepancies and certify appropriate occlusal contact in centric and eccentric relations. The occlusal contacts were verified intraorally for further occlusal adjustment. The patients were instructed in denture cleaning and proper oral hygiene.

###  Construction of 3D-printed complete dentures

**Figure 1:**
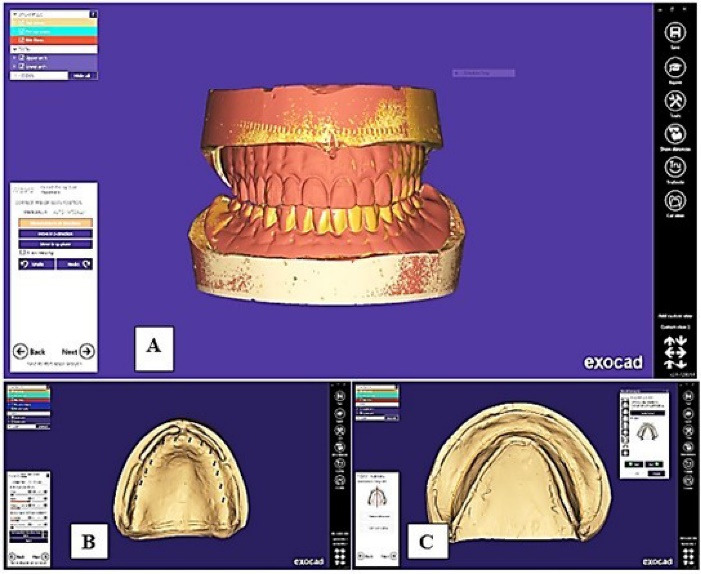
**(A)** The 3D image of the reassembled maxillary and mandibular scanned conventional complete dentures. **(B)** Identification of the anatomic landmarks on the digital image of the scanned maxillary model. **(C)** Identification of the anatomic landmarks on the digital image of the scanned mandibular model

**Figure 2:**
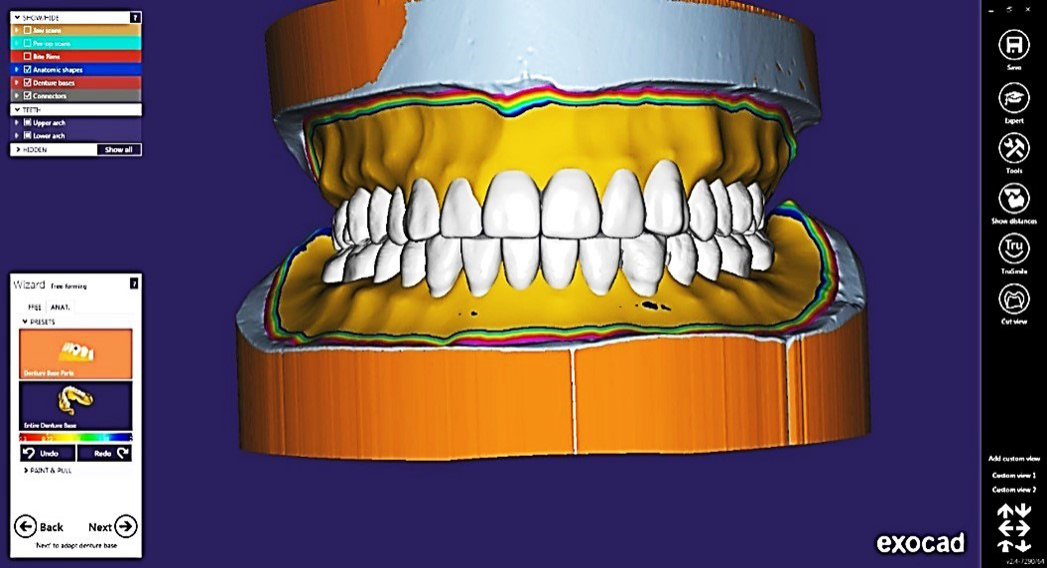
Superimposition of the scanned STL image of the conventional complete denture on the newly designed denture

 Maxillary and mandibular complete denture master casts were scanned using a 3D scanner after being lightly coated with anti-glare spray (Siladent Marmoscan Spray, Basic Ref 250022). The resultant data, in the STL format, were transferred to the CAD-CAM complete denture provider by applying a purpose-built software program (Exocad Dental IDB 2.4 plovdiv7290 [version 2.4 Engine build 7290]). The anatomical landmarks were detected, and the peripheral boundaries were identified on the virtual model in the designing software, which was later used to design the final complete dentures (Figure 1). The scanned STL image of the previously fabricated conventional complete denture was superimposed on the newly designed denture to compare the polished surface, tooth alignment, and form of teeth (Figure 2).

**Figure 3:**
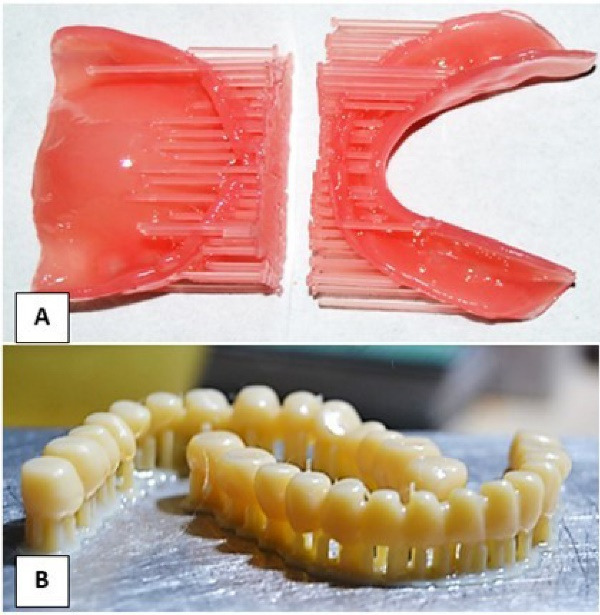
**(A)**The 3D-printed denture bases with supporting posts. **(B)** The 3D-printed teeth with supporting posts

 The denture base shade was selected based on prescription. Wearing proper personal protection equipment, the cassette of the 3D printer (RASDENT 3d Printer) was filled with fresh DENTCA Denture Base II (dimethacrylate-based resins with a photoinitiator). The denture base model STL file was fed into the software. The denture base was oriented vertically on the build platform. Supports were generated around the perimeter of the denture base. The desired slice thickness was selected, and then the printing was started. The denture teeth were printed as one unit with the same method with tooth resin (DENTCA Denture Tooth, Shade A2) after superimposing a scanned STL image of the conventional complete denture on the newly designed denture for comparison of tooth alignment, size, and form (Figure 3).

 The remaining supports were removed, and the denture base was washed with isopropyl alcohol (Isopropyl Alcohol Extra Pure, Alpha Chemical). The printed teeth were glued to the printed denture base. A small amount of shade-matched light-cured adhesive was applied to the tooth sockets, and the teeth were bonded by exposing them to UV light until the teeth were set in position. The final denture was polished with wet polishing sand using a conventional method.

###  Evaluation of denture base adaptation

 Tissue surfaces of denture bases were gently covered with anti-glare spray before scanning (3Shape desktop scanner). The output STL file was imported to the mesh mixer to be flipped. The STL file of the intaglio surface was superimposed on the STL file of the corresponding master cast using surface matching software (Geomagic Control X 64 software), applying the best-fit alignment option.

 The 3D-compare option was applied to assess the adaptation at the whole surface area and at selective seven regions (the upper cast: two regions at right and left hamular notches areas, two regions at the right and left buccal shelve areas, one region at the labial flange, one at the mid-palate, and the last one at the post-dam area; the lower cast: two regions at right and left buccal shelve areas, two at the right and left lingual flanges, two at right and left retromolar pad areas and one at the labial flange).

**Figure 4:**
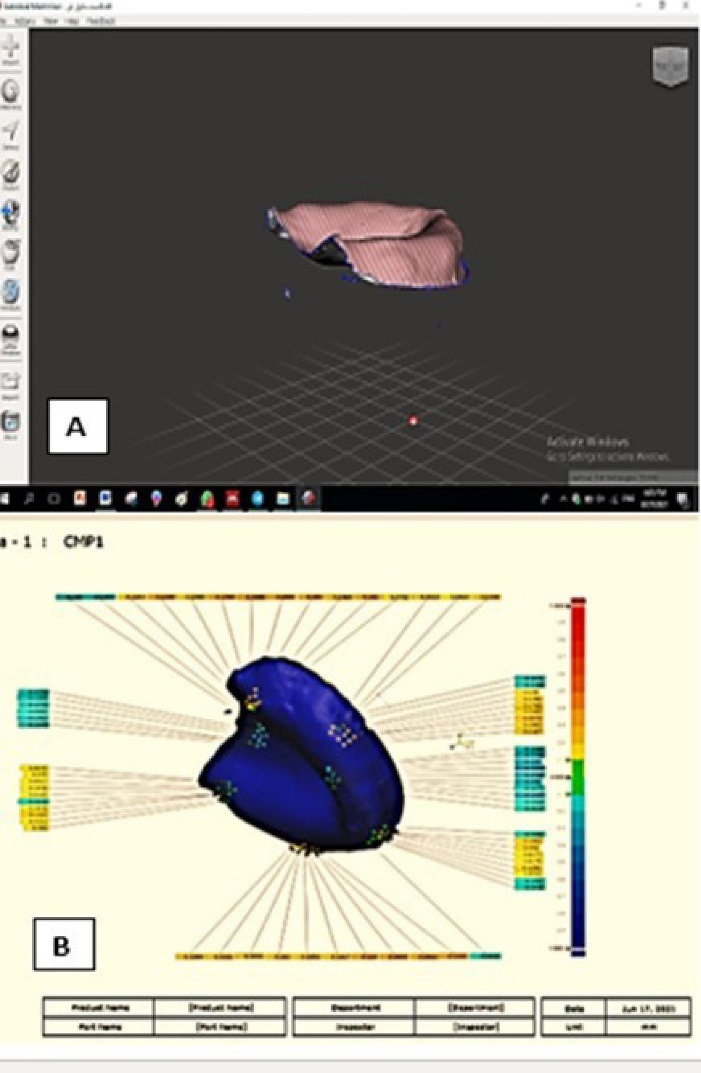
**(A)** Mesh mixer software. **(B) **Geomagic Control X software (measurement of misfit values)

 The denture bases’ adaptation to the casts was reported in numerical values. The positive values implied pressure areas, while the negative values indicated gaps between the denture base and the cast. Moreover, the mean value of the total misfit of the denture base to the cast was calculated. Adaptation was also illustrated in the form of a color scale where yellow to red colors revealed the impingement of the denture base to the cast, while the blue color revealed space, and the green color revealed contact between the denture base and the cast (Figure 4).

###  Evaluation of denture retention

**Figure 5:**
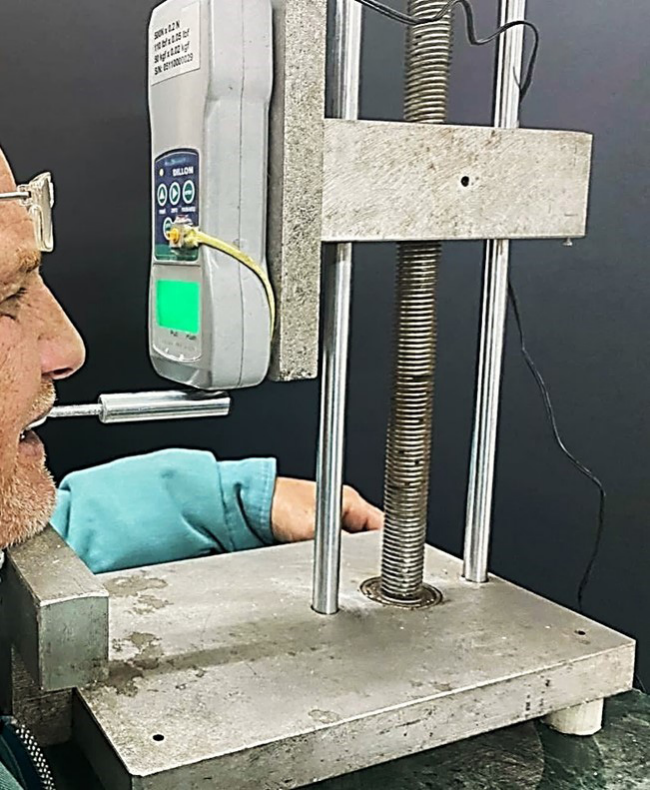
Measurement of denture retention

 The retention of complete dentures for both types was measured as follows:

 Four hooks were connected to the buccal flange using autopolymerized acrylic resin at premolar and molar areas at the same height. The mandibular denture was completely seated intraorally. The patient was asked to set his chin on the device chin-support, with the occlusal plane parallel to the floor. The hooks were engaged to the force meter fork at the pull end. The force meter was used to calculate the pull force required to dislodge the denture base. The same procedure was carried out with the maxillary denture. Five readings were recorded, and the average value was calculated. Data were collected and statistically analyzed to compare the two types of complete dentures (Figure 5).

 The mean values and standard deviation of the measured data were statistically analyzed by statistical software. Repeated-measures ANOVA was used to compare the groups, followed by pairwise comparisons with Bonferroni correction as a post hoc test. The significance level was set at α = 0.05.

## Results

###  Denture base adaptation

Table 1 presents the comparison between maxillary conventional and 3D-printed dentures regarding the mean misfit values of each evaluated region. According to the results, the differences were not significant for all regions and the whole surface area (*P*>0.05). The highest difference was recorded for the left hamular notch area (mean difference = -0.128; higher in the 3D-printed denture). Similar to the maxillary dentures, all the differences in the mandibular dentures were not statistically significant (*P*>0.05). The highest difference was recorded for the labial flange area (mean difference = -0.166; higher in the 3D-printed denture), as shown in Table 2.

**Table 1:** Comparison between maxillary conventional and 3D-printed dentures regarding the mean misfit values of each evaluated region

**Region**	**Denture**	**Mean±SD**	**Mean difference**	**95% CI of the difference**		***P*** value*
				**Lower**	**Upper**	
Labial flange	Conventional	0.011 ± 0.015	-0.068	-0.176	0.041	0.146
	3D-printed	0.078 ± 0.070				
Mid-palate	Conventional	-0.016 ± 0.040	-0.022	-0.329	0.284	0.837
	3D-printed	0.007 ± 0.197				
Right buccal flange	Conventional	0.118 ± 0.033	0.005	-0.084	0.095	0.888
	3D-printed	0.113 ± 0.066				
Left buccal flange	Conventional	0.115 ± 0.048	0.042	-0.098	0.182	0.487
	3D-printed	0.073 ± 0.104				
Right hamular notch	Conventional	0.093 ± 0.031	-0.008	-0.079	0.063	0.790
	3D-printed	0.101 ± 0.049				
Left hamular notch	Conventional	0.119 ± 0.061	-0.128	-0.358	0.103	0.224
	3D-printed	0.247 ± 0.178				
Post dam	Conventional	-0.066 ± 0.078	-0.008	-0.232	0.216	0.931
	3D-printed	-0.057 ± 0.166				
Whole area	Conventional	0.007 ± 0.009	-0.004	-0.025	0.017	0.650

SD, Standard deviation. *Independent *t* test.

**Table 2:** Comparison between mandibular conventional and 3D-printed dentures regarding the mean misfit values of each evaluated region

**Region**	**Denture**	**Mean±SD**	**Mean Difference**	**95% CI of the difference**		***P*** value*
				**Lower**	**Upper**	
Labial flange	Conventional	-0.030 ± 0.023	-0.166	-0.399	0.067	0.110
	3D-printed	0.136 ± 0.149				
Right buccal flange	Conventional	0.085 ± 0.056	-0.013	-0.228	0.203	0.891
	3D-printed	0.098 ± 0.167				
Left buccal flange	Conventional	0.049 ± 0.025	-0.099	-0.244	0.047	0.148
	3D-printed	0.148 ± 0.116				
Right retromolar pad	Conventional	0.040 ± 0.070	-0.007	-0.236	0.222	0.940
	3D-printed	0.047 ± 0.174				
Left retromolar pad	Conventional	0.002 ± 0.034	0.060	-0.227	0.346	0.629
	3D-printed	-0.058 ± 0.232				
Right lingual flanges	Conventional	-0.032 ± 0.087	-0.052	-0.181	0.077	0.363
	3D-printed	0.020 ± 0.059				
Left lingual flanges	Conventional	-0.028 ± 0.060	-0.003	-0.158	0.152	0.967
	3D-printed	-0.025 ± 0.111				
Whole area	Conventional	-0.009 ± 0.018	0.013	-0.016	0.041	0.317
	3D-printed	-0.022 ± 0.014				

SD, Standard deviation. *Independent *t* test.

###  Retention Force

Table 3 shows the differences in retention force values between conventional and 3D-printed dentures (maxillary and mandibular) at each follow-up period and over time. No significant differences were found between the two denture types at each follow-up period (*P*>0.05). Over time, a statistically insignificant increase in the mean retention force values was observed for the two types of dentures (*P*>0.05).

**Table 3:** Differences in retention force values between conventional and 3D-printed dentures (maxillary and mandibular) at each follow-up period and over time

		**3D-printed** **(Mean±SD)**	**Mean difference**	**95% CI of the difference**		***P *** **value**^a^
				**Lower**	**Upper**	
**Maxillary**						
(T0)	121.0 ± 15.0	119.0 ± 13.0	2.0	-22.264	26.264	0.847
(T3)	127.0 ± 11.2	121.0 ± 20.2	6.0	-22.272	34.272	0.622
(T6)	128.3 ± 10.7	121.8 ± 20.4	6.5	-21.715	34.715	0.593
*P*^b^	0.128	0.588				
**Mandibular**						
(T0)	34.3 ± 6.4	33.8 ± 3.9	0.5	-8.642	9.642	0.898
(T3)	35.0 ± 7.3	35.0 ± 4.1	0.0	-10.285	10.285	1.000
(T6)	36.3 ± 7.1	36.5 ± 4.4	-0.3	-10.480	9.980	0.954
*P*^b^	0.071	0.109				

CI: Confidence interval. ^a^Independent *t* test, ^b^Repeated measure ANOVA.

## Discussion

 Keeping denture base deformation during processing as minimal as possible is essential for achieving appropriate adaptation to the mucosa. The degree of this deformation is influenced by the material and thickness of the base in addition to the processing method.^12,13^ Concerning the 3D-printed dentures, the software was used to design the supporting system of the denture base. The orientation of the denture base was adjusted to direct the contact of the supporting arms to the polished surface to maintain the adaptation and precision of the tissue surface.^12,14^ In the present study, the denture base adaptation was evaluated by misfit measurement.^15^ Surface matching and best-fit algorithms were applied to superimpose the images of the scanned denture bases and the casts as carefully as possible, followed by digital measurements. However, most former researchers evaluated denture base adaptation using physical measurements.^16-19^

 The results of this study indicated no significant difference between the two differently fabricated complete dentures regarding adaptation (mean misfit values) of all the evaluated regions of the denture base and the whole surface area. Hence, the first null hypothesis was accepted. This finding agrees with Yoon et al,^15^ who evaluated the trueness of DLP (digital light processing)-produced denture bases to compare the intaglio surface adaptation of DLP to pack/press and milling techniques. They found that concerning trueness, the milled denture base was superior to the DLP one. However, no signiﬁcant difference was identified regarding denture base adaptation regardless of the construction method. They concluded that the intaglio surfaces of DLP and milled denture bases had an accuracy of 100 μm. Although the DLP denture base revealed tissue impingement on the ridge crest, it demonstrated tissue adaptation similar to the milled denture base.

 Concerning the 3D printing procedure, an unpolymerized resin was used for denture base manufacturing, which, once processed, required additional light-polymerization to finish the procedure. Consequently, through the 3D-printing workflow, polymerization shrinkage is hypothetically viable, as the denture bases are not completely polymerized before the last light-polymerization process. Distortion can also occur during demounting the incompletely polymerized denture base from the building platform.^20^

 Despite the satisfactory adaptation values achieved in the group of conventional dentures in the current study, this technique has numerous drawbacks. Factors such as the difficulty of handling, time-consuming processes of waxing-up, investing, and packing, together with the distortion of heat-polymerized PMMA, may reduce denture base adaptation.^12,13,21^

 Reasonable and equivalent retention values were detected in both conventionally fabricated and 3D-printed dentures; however, there was an insignificant increase in retention over time. Accordingly, the second null hypothesis was accepted, too.

 The observed acceptable and equivalent retention values of both denture types could be attributed to the documented reasonable adaptation of denture bases.^22^ The detected increase in denture retention over time highlights the influence of patient neuromuscular coordination established with function.^23^

 Faty et al^24^ assessed the adaptation and retention of milled and 3D-printed denture bases and compared them to conventional ones. The results showed significant differences between the three groups. They found that the greatest values of adaptation and retention of denture bases were observed in group II (milling group).They concluded thatmilled denture bases exhibited superior adaptation and retention than the traditional heat-polymerized and 3D-printed denture bases. Although the 3D-printed denture bases revealed superior adaptation, their retention was similar to conventional heat-polymerized denture bases.

## Acknowledgments

 None.

## Author Contributions

**Conceptualization: **Radwa Mohsen Kamal Emera.

**Data curation: **Mohamed Shady, Mahmoud Almabrouk Alnajih.

**Formal Analysis: **Mohamed Shady,Mahmoud Almabrouk Alnajih

**Investigation: **Mohamed Shady, Mahmoud Almabrouk Alnajih

**Methodology: **Radwa Mohsen Kamal Emera.

**Writing – original draft: **Radwa Mohsen Kamal Emera, Mohamed Shady, Mahmoud Almabrouk Alnajih.

**Writing – review & editing: **Radwa Mohsen Kamal Emera, Mohamed Shady, Mahmoud Almabrouk Alnajih.

## Funding

 Self-funded.

## Ethics Approval

 The study protocol was approved by the Institutional Ethics Committee of the Faculty of Dentistry, Mansoura University, Egypt (Code: A04031219).

## Competing Interests

 The authors declare no conflict of interests related to the publication of this work.
